# Homestay accommodation as care work: a case study of private accommodation for refugees from Ukraine in Switzerland

**DOI:** 10.3389/fsoc.2025.1571633

**Published:** 2025-06-30

**Authors:** Eveline Ammann Dula, Gesine Fuchs

**Affiliations:** ^1^School of Social Work, Bern University of Applied Sciences, Bern, Switzerland; ^2^School of Social Work, Lucerne University of Applied Sciences and Arts, Lucerne, Switzerland

**Keywords:** homestay accommodation, civil society, care, refugees, gender, Switzerland

## Abstract

In this paper we conceptualize homestay accommodation as care, through the feminist lens of Joan C. Tronto’s seminal works on the subject, based on a qualitative and quantitative survey of Ukrainian refugees in Switzerland. We used Tronto’s definition of care as an analytical framework to analyze care providing, giving, and refusing as negotiation processes in the context of unequal power relations between hosts and refugees, but also between civil society and the state. We identified a practical dimension of care, seen through the way hosts take care of the wellbeing of refugees. This form of care requires a lot of planning, coordination and organization, but also emotional engagement. For hosts, this means a large mental load, feeling responsible and providing this support in addition to their regular work and family life. On the other hand, refugees are not only receiving care, but also providing care or refusing care for different reasons. These negotiations can lead to conflicts and are embedded in power relations between hosts and refugees. Hosts often took on tasks that should actually be the responsibility of the authorities. The provision of private accommodation for refugees can be seen as an act of civil society to support the authorities, thus improving their capacity to accommodate refugees, often in line with official migration policy by incorporating expectations regarding the integration of refugees. However, there were also cases in which the host criticized state policy and showed solidarity with the refugees. The care perspective allows us to analyze the power relations that permeate relationships between hosts and refugees. We argue that the dynamics of private accommodation reflects or confirms current power relations between refugees and the host, but also has the potential to shift power relations between the state and civil society—as persons offering homestay accommodation address conflicts about the provision of care at the institutional and political level. It is in this way that the transfer of responsibility from the state to civil society is being questioned. Private accommodation has therefore the capacity to build forms of solidarity between refugees and civil society, linked to different forms of care providing and care needs.

## 1 Introduction

After the start of Russia’s full-scale invasion of Ukraine in 2022, refugees fleeing to European countries met a great wave of solidarity (OECD, 2022). By the end of 2022, i.e., the period of our research, around 75,000 people from Ukraine had applied for protection in Switzerland. While around 60 per cent of refugees initially lived in private accommodation, in May 2023 this figure had fallen to just under a third (SEM, 2023). Without private engagement the authorities would have been overwhelmed by the high numbers of people fleeing from the war. For the first time in recent history, private accommodation was a key element in the official refugee reception policy.

Since the mid-2010s homestay accommodation for refugees in Europe has become not just an individual phenomenon, but a part of civil society engagement and state-supported projects (Bassoli and Luccioni, 2024). Homestay accommodation fits within a tradition of civil society engagement and unpaid care work in the field of migration. It gained salience and visibility with “the summer of migration” in 2015 and in some cases led to wider solidarity movements in Europe (Bassoli and Luccioni, 2024, p. 1534). At the same time, academic research on the topic has also increased, as a literature review study from Bassoli and Luccioni (2024) shows, cowering studies conducted mostly between 2015 and 2020 in mostly European countries (Austria, Belgium, Finland, France, Germany, Italy, the Netherlands, Switzerland and the United Kingdom, p. 1539). Where socio-demographic data is available, previous studies have shown that gender and class are relevant to the decision to offer private accommodation: hosts tend to be wealthy, well educated, national citizens, and more often female. They live in different types of households and are often middle-aged (Bassoli and Luccioni, 2024, p. 1547). Thus, gender and class are relevant also for the reception of Ukrainian refugees, as volunteer work in the care sector in Switzerland is also more likely to be carried out by women with a higher socio-economic status (Bundesamt für Statistik, 2021).

In our collaborative research project “name withheld” we explored the potential of homestay accommodation^1^ for the social integration of refugees and the fulfilment of their housing needs. We also explored the reasons behind the remarkable engagement and support from society.

The issue of care—a job mainly ascribed to and done by women* (Bomert et al., 2021) or femininity in general (Riegraf, 2018)—emerged as a central theme for hosts and refugees alike in both our qualitative and quantitative data. This care work is relevant in its practical and emotional aspects.

In this paper we conceptualize homestay accommodation as care, using the feminist lens of Joan C. Tronto’s seminal works on the feminist ethics of care. This care perspective allows us to analyze the power relations that have been found to permeate relationships between hosts and refugees (Bassoli and Luccioni, 2024). By analyzing the relation between refugees and host as care and by examining its power dimensions we will contribute to overcoming the limitations identified in the literature by Bassoli and Luccioni, who found that studies often overlook the asymmetry between hosts and guests.

Firstly, we introduce our research project, present the basic data and findings, and embed the results in the context of Swiss civil society. Then we go on to introduce the concept of care, based on Joan Tronto’s. Finally, we analyze and discuss our findings on different kinds of care in homestay accommodation.

## 2 The study, data, and methods

### 2.1 Data and methods—interviews and online survey, field access, coding and analysis

The project “name withheld” investigated how refugees and hosts experienced private accommodation, and whether and in what way private accommodation facilitates the arrival of refugees and promotes social integration. It consisted of a mixed-method design, based on both qualitative and quantitative data.

*Quantitative data collection:* between October and December 2022 we conducted an online survey in collaboration with Swiss Refugee Council (SFH), resulting in 986 responses from 19 of 26 Swiss cantons, from hosts with one or more refugees from Ukraine, staying for at least 4 weeks. We asked the hosts for information about their living situation; the people they had taken in and the support they received; living together and contacts with the authorities; and finally, what they considered important for social integration. In addition to closed questions, we also asked open questions that provided further information on these topics. Open-ended questions in quantitative surveys are usually used to obtain extra information, for example to explore new aspects of a topic or broaden its spectrum (Züll and Menold, 2019, p. 855) and to mitigate the risk of social desirability of the answers (Wagner-Schelewsky and Hering, 2019, pp. 788–89). This survey provided us with a unique and rich dataset, with the open answers giving us an excellent insight into experiences (verbalized) everyday knowledge, and wishes or demands vis-à-vis politics that can also be quantified, with all due caution.

*Qualitative data collection:* to get an initial overview we started with informational interviews with the authorities and three NGOs, in two cantons in German-speaking Switzerland. They were asked about the preparation, organization, and concrete activities involved in managing the arrival of the refugees; and also, to assess the importance of civil society involvement.

In order to contribute to a better understanding of the perspectives of refugees and the persons providing homestay accommodation, we conducted narrative interviews with these two groups between August 2022 and March 2023. We spoke to a total of 12 refugees and 12 hosts in German-speaking Switzerland. We were able to include both urban and rural living situations. The private placements lasted from between 6 weeks to around 6 months. The placements ranged from single persons to several members of a family (maximum of four people). Placements were made directly with private individuals: partly via specialized organizations, partly via a university. In the sample of refugees, it is noticeable that they were all women who came to Switzerland alone or with their children. This reflects the gender ratio of refugees from Ukraine in Switzerland. In December 2022, 65% of the 62,820 persons of working age with protection status S were women.^2^

To address ethical and methodological issues involved in interviewing people who might have experienced trauma or repeated injunction to tell their stories (Bassoli and Luccioni, 2024, p. 1550) we decided to recruit Ukrainians to our research team so that the interviews could be conducted in the refugees’ native language. To this end we engaged Ukrainian students and a member of staff to conduct and transcribe the interviews.

In the narrative interviews (Schütze, 1983), we asked open-ended questions, allowing the interviewees to respond by sharing their experiences in a story-like format. We asked them to tell us more about their experience of private accommodation, how it came about, and what it was like. This was followed by questions about living together, support and moving out, which were laid down in an interview guide based on housing needs (Deinsberger-Deinsweger, 2017; Leising, 2002). These interviews were transcribed, translated where necessary, and coded with the help of MAXQDA both inductively with the regard to forms and needs of care, and deductively with regard to motives and housing needs, and then analyzed (see Ammann Dula et al., 2024).

### 2.2 Overview of key results^3^

In March 2022, Switzerland activated Status S for people fleeing Ukraine shortly after the European Union invoked the Temporary Protection Directive (2001/55/EC). The two regulations are similar. Under Status S, refugees are not required to undergo a formal asylum procedure, nor are they formally recognized as refugees. Those with Status S obtain a residence permit and permission to work, and family reunification is possible. This distinguishes Status S from other refugee regulations. However, Status S only provides temporary protection, which can hinder social integration. Refugees with Status S are entitled to social assistance, which is significantly lower than that provided to locals.

The online survey showed that hosts tended to be economically well-off, middle-aged people with plenty of living space, mainly located in urban areas. Households constituted of families, single people, or flat-sharing communities. Two-thirds of the hosts who answered our survey were female (one person identified as non-binary). Three quarters of them were house owners, a proportion which is twice the Swiss average.^4^ On average, each household hosted two people. Of the people hosted 70% were female and 30% male, one third was under-age, and barely 5% of people were aged 65 or more.

In the qualitative interviews, both hosts and refugees described the homestay accommodation experience as positive, especially in the initial phase: it promoted orientation, support, and safety. However, housing needs can only be put on hold for a certain period of time. The biggest challenge for both sides was maintaining privacy in the long run, and for the refugees the feeling of not wanting to be a burden. Issues identified were the lack of space and opportunities for retreat, rest, and relaxation. In connection with time rhythms and the organization of the use of space, hosts and refugees needed to organize and negotiate their arrangements, preferably at an early stage in the homestay experience.

Finding and speaking a common language is central to living together, especially when bathrooms and kitchens are shared. The online survey showed that almost all used translation apps, over 50% of hosts communicated in English, and Russian was used by almost 13%. Apart from language, successful communication also needed mutual openness, respect, and a willingness to exchange ideas and clarify expectations and needs.

Our quantitative data showed that following a period of homestay accommodation refugees often found their own accommodation and/or a job through the support of the hosts; they benefited in this way from practical information and concrete support from the hosts. In this sense homestay accommodation can boost social integration in a way not possible in shared accommodation (Baier et al., 2022), but more research is necessary to observe how this evolves over time. The State Secretariat of Migration has commissioned a report that includes a cost–benefit analysis of homestay accommodation for refugees.^5^

With our quantitative data we can confirm that “class” is a relevant factor for the hosting of Ukrainian refugees: the availability of sufficient living space and time is often a central motivation for taking in refugees, regarding the ability to provide care for refugees in the form of homestay accommodation. In our quantitative and qualitative sample, both men and women took over care work in the role as hosts offering private accommodation for refugees. However, two thirds of respondents in the online survey and most of the interviewees were female, reflecting gender roles in caring. The issues of how care is recognized, provided, negotiated, and received was central in our data. Moreover, especially hosts discussed the division of the provision of care between the state, civil society, and the private sphere.

## 3 Civil society, the state and migration policy in Switzerland

### 3.1 Functions of civil society

Civil society can be defined as a public sphere between market and the state, where people voluntarily act together in initiatives, associations, clubs or social movements to pursue shared interests (Geißel and Freise, 2016, p. 528; Adloff, 2005, pp. 25–29). Several well-developed theoretical traditions assign protective, mediating, socializing, interest aggregation and contentious functions to civil society. In a liberal understanding following John Locke, civil society protects citizens from unjustified state intervention and interference, particularly in their civil and political rights—such functions are performed today by numerous civil and human rights organizations. As an area of social self-organization, civil society performs a complementary mediating function between the state and citizens, in which independent bodies limit political power and contribute to the formation of a common will. According to Alexis de Tocqueville, free associations in civil society are schools of democracy, in which civic virtues such as tolerance, trust, and willingness to compromise are practiced and normatively anchored; yet contemporary empirical findings question direct implications of internal organizational structures on democratic behaviour (e.g., Hinterhuber, 2012). Civil society has an important communicative function in that it offers citizens space for debate, consultation, and participation in democratic decision-making and influence on the economic and political sphere. In particular, interests that are difficult to organize or disadvantaged can be integrated into this debate via civil society, thereby creating more democracy (fundamental: Cohen and Arato, 1992). Following on from this, a Gramscian view of civil society as a place of struggle for hegemony is important (Brighenti, 2016; Martin, 2023): civil society is a place of conflict and struggle for the inclusion or exclusion of marginalized social groups in a democracy (Hinterhuber, 2014, pp. 8–9).

It is not only political activities in the narrow sense that are relevant; Gramsci deserves credit for drawing attention to the importance of intellectuals as actors in establishing a new worldview. Civil society thus also reveals itself as an ambivalent place of dominance and resistance (Schade, 2002, p. 15), which is not only a refuge for “the civil,” but also a place for social groups, movements, or ideologies that can be authoritarian, violent, racist, or sexist. Social cohesion or the production of social capital is the focus of sociological perspectives. According to Putnam (1993), social networks strengthen norms of reciprocity and social trust. These ties between individuals help build mutual trust, which is important for social cooperation in general as trust fosters cooperation and mutual aid (Filsinger and Freitag, 2020). However, civil society is also characterized by hierarchies and inequality when it comes to reputation, influence and recognition. The internal structures of initiatives and organizations, as well as the profile of volunteer work, are all gendered (Hinterhuber, 2014).

### 3.2 Civil society and migration in Switzerland

Switzerland has strong a strong civil society that controls and complements state activities and produces social capital (Helmig et al., 2011). State-society relations in Switzerland can best be characterized as republican: civil society participates in debate of public affairs and simultaneously implements public services (Nadai, 2006, p. 344; Freitag et al., 2019). Strong federalism and subsidiarity are central identity-forming principles. Subsidiarity means that (a) central authorities should perform only the tasks that cannot be performed by lower local levels and (b) people are responsible for themselves, and only if they cannot manage on their own does the state step in to support them (Wincott, 2018; Studer, 2020). Subsidiarity is central in social service provision: in Switzerland, a welfare state latecomer, private social security and social services (for-profit and non-profit) often preceded state provisions. Services, from counselling to institutional care and unemployment funds, are still partly provided on a subsidiary basis, but are now financed by the state and are organized via so called service agreements (Canonica, 2019). The autonomy of civil society has never been questioned, and the government has a positive stance toward NGOs and their delivery of services (Helmig et al., 2011, pp. 22–23).

Subsidiarity also plays a central role in migration policy. Large NGOs like Caritas, the Red Cross or the Salvation Army provide services in refugee care, manage collective accommodation, and “return counselling” (see also Schilliger, 2023). Additionally, NGOs can often rely on the volunteer work of their members, e.g., in mentoring or language training. Some commercial players like ORS^6^ are also contracted to run services. This results in a diffusion of responsibility from the state toward organizations or firms where the state tries not to be held accountable e. g. for poor service quality (Alberti, 2022). It has been said that the implementation of state policies through civil society organizations can weaken critical voices, as this gives policies a more “human face” and strengthens the acceptance of increasingly restrictive migration regimes (Andersson, 2017). Indeed, in international comparison Swiss civil society is not very contentious and protests against migration policies are rare (Ruedin et al., 2018).

In Switzerland 30% of men, as compared with only 18% of women are active in civil society organizations, i.e., in “institutionalized volunteer work” (Bundesamt für Statistik, 2021). Women are more active in church and charitable organizations, whereas men are more often in leisure clubs, political office or political parties (ibid.). Informal volunteer work is predominantly unpaid care work, here defined as engagement outside one’s own household. According to current statistics, women dominate here: every fourth woman, but only every eighth man, cares for children; similar proportions are found in care for the elderly, the ill, or people with disabilities. Finally, according to the statistics, the following persons are more likely to volunteer (a) people who have a higher level of education (b) live in German-speaking Switzerland and (c) live in in rural areas (Bundesamt für Statistik, 2021, p. 4).

Activist work for refugees in Switzerland has grown since 2015 and corresponds to Europe-wide developments in civil society which, according to Pries (2019, p. 2) compensates for the failure of states. This includes initiatives for illegalized persons (including undocumented migrants and rejected asylum seekers) which offer political and practical support for everyday life, education, and (legal) advice (Kilic and Kilic, 2023), as well as a commitment to the private accommodation of refugees.

Such “off-loading” of state tasks to the private sector has been criticized as exploitative and as the replacement of rights with charity (e.g., in language classes, legal aid and medical care) Van Dyk and Haubner (2021) claim for Germany a “community capitalism” that exploits unpaid care work to fill structural gaps in public infrastructures. Comprehensive research on this phenomenon in Switzerland is still lacking. This civil society commitment contrasts with sustained initiatives by right-wing parties, who advocate for more restrictive access to asylum (Prodolliet, 2019, p. 7) and distinguish between “genuine war refugees” and “economic refugees.”

The engagement of Swiss civil society with Ukrainian refugees takes various forms, ranging from the implementation of state policies to self-organization. Well-established organizations such as Caritas, HEKS (Swiss Church Aid) and SAH (Swiss Workers’ Relief Organization) provide care and support for refugees in the form of education, language classes and counselling, through service agreements with the federal state and cantons. The Swiss Refugee Council focuses more on advocacy. As it had implemented a homestay accommodation project in 2016/17 with Syrian, Afghan and Eritrean refugees, it could draw on experiences and good practices to promote it also for Ukrainians from 2022 on.

At regional and local levels, numerous initiatives and organizations offer meeting venues and social, cultural and legal support. They receive funding from a variety of sources, including donations, sponsorships, project funding and small service contracts, and rely on volunteers (examples: hellowelcome.ch, helpnet-frutigland.ch). Some cantons have set up coordination points for volunteer work which bring offers for Ukrainian refugees and interested parties together. Finally, there are self-organized initiatives such as Prostir (prostir.ch) in Lucerne. Founded by Swiss locals, the organization is now mainly run by Ukrainians and its activities are open to all refugees.

Overall, civil society has stepped in to prevent state structures from becoming overwhelmed. This commitment went far beyond what is usually considered support for refugees. Comments on integration in the online survey show that people with a wide range of political views were involved: some demanded compulsory work in exchange for social welfare and invoked the “bogus refugees” discourse. However, left-wing slogans such as “no borders” were also present, and hosts demanded that status S be extended to all refugees.

Several respondents had previously hosted refugees. In one town where some of the interviews were conducted, a refugee initiative founded in 2015 was quickly reactivated to accommodate many Ukrainians.

Overall, Switzerland shows that civil society plays a central role in the care of refugees. On the one hand, civil society complements the state and offers a great deal of support, especially in the areas of language and social integration. However, it is also taking on more and more tasks that are no longer being funded by the state due to increasingly limited state resources, resulting from politically motivated cutbacks. The Swiss state’s commitment is characterized by federalist structures, which means that the design of support for refugees and their accommodation varies greatly from canton to canton, and sometimes even at the municipal level.

## 4 Theoretical perspective: cycles of care according to Tronto

Care has been named a “key feminist concern” because its provision remains gendered, which has profound implications on what men and women can do (Himmelweit and Plomien, 2014, p. 446). Tronto and Fisher (1990) developed their concepts of care in the context of feminist care ethics (Norlock, 2019). In general, care is an encompassing activity that includes:

*“Everything we do to maintain, continue, and repair our ‘world’ so that we can live in it as well as possible. That world includes our bodies, ourselves, and our environment, all of which we seek to interweave in a complex, life-sustaining web.” (Tronto and Fisher, 1990, p. 40)*

Within feminist ethics a normative value is assigned to care, and theorists insist that “political subjects are not independent, but fundamentally (inter)dependent, enmeshed in complex networks of relation necessary for survival” (McAfee and Howard, 2023, p. 2.5; Razavi, 2007). The unequal gender distribution of care produces and reflects other structural inequalities, as social reproduction theory as well as Black and decolonial critics of care theory have underlined (Arruzza et al., 2019; Beier et al., 2023).

For the purposes of this paper, Tronto’s conceptualization is helpful as it includes both interpersonal relations in, and political implications of the provision of care. Care conflicts in homestay accommodation arise between hosts and refugees, but also between hosts and the state. The distribution of care between households (unpaid work), the community (non-profit), the state and private entities (for profit) is contested (cf. Razavi, 2007, p. 21). Contrary to what occurs in other contexts, care in homestay accommodation is, for once, provided mainly from “above” (the rather well-off natives) to the vulnerable people, i.e., the refugees.

Tronto and Fisher not only place care on an interpersonal level and as individual responsibility, but also conceptualize it as a fundamental social and political phenomenon, highlighting the interconnectedness of lives and environments. Care can occur in a variety of settings: in households as well as in markets and institutions. Because care has been associated with the household (and the women in it) it has been greatly undervalued in a world that is seen to be divided between public and private spheres (see Squires, 2003; Nowotniak, 2024 for an account on this “great dichotomy” in Western thought).

We argue that homestay accommodation can be conceptualized as form of care. It is a social and political phenomenon of providing support for refugees at the intersection between civil society and the state, namely in the private sphere: hosts are opening their private sphere to persons they do not know and providing more than just shelter.

According to Tronto (1998, S.16), care has a dual meaning, namely as a ‘mental disposition of concern’ and an actual practice.

*“An analysis of care can provide us with a useful guide for thinking about how we do our particular caring work and its ethical dimensions- the way in which care is related to what we know about how to live a good life. Such a process can also provide us with a framework for political change.”*

Tronto has identified five phase of care as follows (see Tronto and Fisher, 1990; Tronto, 1998; Tronto, 2013):

- Caring about involves becoming aware of and paying attention to the need for caring. To care genuinely about someone, some people, or something requires listening to articulated needs, recognizing unspoken needs, distinguishing between and deciding which needs to care about. It requires attentiveness, being able to perceive needs in oneself and others, and perceiving them with as little distortion as possible, which could be said to be a moral or ethical quality (Tronto, 1998, p. 16).

In terms of private accommodation, this means that hosts are aware of the actual needs of refugees and decide accordingly which needs to address. This requires knowledge and experience in dealing with flight, trauma and transcultural skills. A major challenge is dealing with language and communication, which is made easier when people have a similar level of education and interests.

- Caring for is the phase in caring when someone assumes the responsibility of meeting a need that has been identified. Simply seeing a need for care is not enough to make care happen; someone has to assume the responsibility for organizing, marshaling resources or personnel, and paying for the care work that will meet the identified needs. The moral dimension of caring for is to assume, and to take seriously, responsibility (Tronto, 1998, p. 16).

Regarding homestay accommodation, this means that it is not enough to just be aware of the needs of refugees, but this should result in a corresponding action or activity. This might refer to the hosts providing support responding to refugees needs, but also to the state, who should not only identify needs of refugees, but also provide the resources necessary to care for them—either directly or via specialized organizations and/or civil society.

- *Caregiving*. This phase is the actual material meeting of the caring need. Caregiving requires that individuals and organizations perform the necessary caring tasks. It involves knowledge about how to care. Although we often do not think of it this way, competence is the moral dimension of caregiving. Incompetent care is not only a technical problem, but a moral one (Tronto, 1998, p. 16).

Caring for refugees requires resources like time and space, competences like knowledge of existing services, and the time and resources to make them accessible. A moral dimension of caregiving is that of making sure that hosts are able to provide the necessary support.

- *Care receiving*. This phase involves the response of the thing, person, or group that receives the caregiving. Whether the needs have been met or not, whether the caregiving was successful or not, there will be some response to the care that has been given. Care receiving requires the complex moral element of responsiveness. Responsiveness is complex because it shares the moral burden with the person, thing, or group that has received the care, but it also involves the moral attention of the ones who are doing the caring work and those who are responsible for the care. In a way, since any single act of care may alter the situation and produce new needs for care, the caring process in this way comes full circle, with responsiveness requiring more attentiveness (Tronto, 1998, p. 16).

Refugees might respond in different ways to the care that has been given. As we will show in our results, it can be a moral burden to receive the support of private persons and live in their private space, something linked to the general situations of refugees, of losing the autonomy to take care of themselves.

In Tronto’s more recent theoretical texts (2013), she adds “caring with” as a fifth cycle of care that builds on the expectations around the “feed-back loop” that works among the four phases: “When care is responded to, through care-receiving, and new needs are identified, we return to the first phase and begin again.”

- Caring with refers to processes that results over time, when people come to expect that there will be ongoing engagement in care processes with others, that need trust and solidarity, as people realize that they can rely upon others and come to understand that they are better off engaged in such processes of care together rather than alone (Tronto, 2013).

When it comes to accommodation by hosts, these forms of solidarity and trust networks can develop between refugees who begin to organize themselves and establish forms of support networks among themselves. Hosts can also organize themselves and establish support networks, or networks can even develop between refugees and hosts, for example through accommodation in host families, which in turn can lead to the formation of neighborhood organizations. Regarding Tronto’s (2013) definition, time appears to be a relevant factors, and the central question arises as to how long these support processes must last in order to lead to continuous engagement, and how continuous engagement can be defined.

According to Tronto, providing care is also related to power relations and conflicts, which are particularly relevant in the context of homestay accommodation for refugees.

- *Power relations occur*. When somebody is an employee doing care work or when caregivers have abilities the care receivers must rely on; and of course, care relations can be abused by any party (Tronto, 1998, p. 17). One should ask how needs are understood, which forms of power and privilege reside in the provision of care? And which “othering” takes place in care provision for refugees? (Tronto, 1998, p. 18).

The relation between hosts and refugees is power laden because of their differing legal and economic status in Swiss society. Being a refugee is related to a loss of autonomy and increases dependency on the state regarding rights and duties. Housing, employment, and possibility of financial support are highly regulated and might differ depending on the municipality or canton. In contrast, hosts are often in a privileged situation as not only our study, but also previous studies, show (Bassoli and Luccioni, 2024, p. 1547).

*“Any situation of hospitality creates an asymmetric relationship between the host, who is at home, and the guest, who is given a precarious right to stay (see also Derrida et al., 1999, 2000; Gotman, 2001). In a few cases, to reduce refugees’ dependence on their hosts, hospitality took the form of a rental relation; the rent may be financed thanks to social benefits (Brotschi, 2017), sometimes completed by crowdfunding (Zenkl, 2017). In other contexts, guests performed household chores (cleaning, cooking, etc.) and offered their hosts informal financial contributions, as counter-gifts to reciprocate (Komter and van Leer, 2012, pp. 18–19). However, in some situations, a persistent feeling of a “debt of gratitude” (Merikoski, 2019, p. 123) made co-habitation difficult.”* (Bassoli and Luccioni, 2024, p. 1549)

According to Tronto (2000), conflicts are another important aspect regarding care ethics, as there are more care needs than can ever be met.

- *Care is fraught with conflicts*. Caregivers also must balance their own needs with the needs of others. On the institutional level, conflict arises between different groups or clients—on the political level conflicts exist over the recognition of needs of different individuals or groups who voice—or cannot voice—their demands. The discursive construction of needs takes place in the political sphere and is a process of contestation and (counter)hegemonic discourses, and its analysis is a core issue of feminist political science (Ackerly and True, 2018; Bacchi, 1999; Fraser, 1993). Therefore, it is important for democratic practice to actively engage in caring relationships and activities in order to practice the moral skills (such as attention, responsibility, empathy and self-reflection) that make up life (Tronto, 2000, p. 37).

With regard to refugee accommodation, a possible source of conflict for the host is trying to reconcile their own needs and those of the refugees, especially when the period of accommodation lasts longer than several months. On an institutional level, the differences between refugees with differing status and rights and possibilities, depending on their origin, might be a source of conflict, as well as between refugees in different accommodation settings, as the care provided in collective shelters might be different from the support received in private accommodation (Baier et al., 2022). These differences have been articulated by different actors from civil society, especially regarding the new S status for Ukrainian refugees and compared to other forms of status for refugees of other nationalities. Contrary to other studies on homestay accommodation for illegalized refugees or from other regions (Gunaratnam, 2021; Merikoski, 2021; Kekstaite, 2022), we do not find any fundamental criticisms of the state or state policy among the hosts.

## 5 Findings: homestay accommodation as care work

An initial inductive analysis, based on the grounded theory (Glaser and Strauss, 1999) methodology of qualitative interviews with refugees as well as the hosts, has shown that the topic of care plays a central role, and manifests itself in different ways. We found care as a central issue in the open survey answers as well and structured the findings according to Tronto’s definition of care.

### 5.1 Caring about—realizing the need of care

As a first phase of care, is important “becoming aware of and paying attention to the need of caring” (Tronto, 1998, p. 16). This implies first the willingness to offer homestay accommodation, but then also the ability to pay attention to the effective needs of the hosted refugees.

#### 5.1.1 Realizing the needs of refugees

To provide care in homestay accommodation, hosts have first to recognize the need of refugees for accommodation. The great willingness of people to take in refugees in 2022 points to a high awareness of the need for care. People were able and willing to realize this need more often than in previous crises. In the survey we asked for motives for taking people in (Table 1). Furthermore, we gave the opportunity for an open answer, and we found wide agreement on the empathetic impulse of caring about.

**Table 1:** Motives to take refugees in (*N* = 986).

Motives	*N*	Percent
Need to contribute in times of crisis	758	76.9%
Human compassion	735	74.5%
Showing solidarity with Ukraine	627	63.6%
Gratitude, a sense of giving because you are doing so well	627	63.6%
To make it easier for refugees to integrate in Switzerland	440	44.6%
I have free living space and want it to be used	438	44.4%
Sense of moral or religious obligation	244	24.7%
Being a good example to my children	198	20.1%
Getting to know another culture	156	15.8%
Other	75	7.6%
Personal connection to the issue of flight	74	7.5%
Personal connection to Ukraine	43	4.4%

Source: calculated from online survey.

Becoming aware of the need to provide shelter for the refugees from Ukraine was the main motivation for offering homestay accommodation. The hosts expressed the desire to make a contribution during the crisis, showed empathy or expressed gratitude for living in such privileged, peaceful conditions. About 8% added open answers: one person mentioned becoming a host when they learned about the unacceptable conditions in some collective shelters. Some recalled family memories of flight and refuge during the Second World War:

*“My mother and her family were bombed out during the Second World War and were able to live with a farming family until the end of the war*.”

Finally, there was also “caring about” as means to fight their own helplessness: “*If I cannot go and shoot Putin, and cannot DO anything else, then at least I can do this*.”

The motivations of the hosts indicate a perception of the general needs of the refugees from Ukraine. However, caring about requires also listening to articulated needs or recognizing unspoken needs, which can be a challenge. The interviews showed that fulfilling the real needs of care was a challenge for hosts and refugees, although most of the hosts were very committed to providing care.

Unfortunately, there were also cases where the hosts did not recognize the needs of care or were not willing or able to respond to them. This power imbalance—as refugee you cannot force hosts to care –can lead to the termination of private accommodation, as the example of Mr. Hunn shows. Mr. Hunn, a host from the qualitative sample, was not willing to look after an elderly woman when her son wanted to go away for the weekend. In the survey we also found three cases, where hosts explicitly did not want to care for children when the mothers went somewhere on their own (ID 1471, 821, 638).

*They called me and I immediately agreed to host a woman that evening. However, after that, no one got in touch to explain how to proceed. (…) As I had so many obligations during the day, I found myself in a situation that was becoming more tense by the day. She wanted me to go with her to some place she’d heard about in city X, she wanted me to give up my holiday because she didn’t want to live in the flat on her own, etc. It was becoming complicated for the whole family, to say the least. (ID 1206)*

This is also reflected in the occasional disappointed comments in the online survey. Hosts sometimes had an attitude of entitlement, which in itself signals power over the refugees. Some had a clear idea of care, but also of consideration, which was presumably not discussed in advance. For example, the expectation of shared meals, the completion of a language course and information about when and where the refugee was going (e.g., ID 529). More common (around 5%) were complaints about too little care, in the form of housework, done by the refugees.

### 5.2 State authorities and the host’s need for care

But not only the refugees need care, also the hosts mentioned their needs of support. In the survey several hosts reported that they had underestimated the burden, and that self-protection was necessary when dealing with traumatized people. Some hosts named the need to take care of themselves as a mental load that was clearly noticeable in the long term (ID 698). Particularly striking are experiences where the need for administrative support and contact (attempts) with the authorities came at the expense of the person’s own employment:

*“I have had to invest an extreme amount of work because the municipality has commissioned refugee organization X, which acts in a very dismissive, sometimes beyond unfriendly manner and does not take care of very important information clarifications - despite being responsible. There are only discussions between X and refugees, refugees and us, us and the municipality, the municipality and X. As a result, nothing worked out until I put in a lot of time and effort (which I can’t really afford because I have had to neglect my professional work as a result). School enrollment, language courses, bus ticket for trips there, conversation courses, job search, clarification of the legal and financial conditions for working and much more.” (ID 789)*

In general, institutional support from the authorities in the first months was scarce, or absent altogether, and thus increased the mental load.

*“we actually took over the work of a social worker for 3 months, we hardly get anything from the authorities.” (ID 304)*

As hosts were on their own, they searched for relevant information, some contacted other hosts, organized themselves or asked around in their own network. Ms. Cesare, for example, found an online chat for hosts, which she used to obtain useful information:

“*and nobody tells you: you have to register there, and you have to register there. I always wrote a list myself, and later I joined a chat of ‘hosts’. Whenever there was any information, someone posted it. That always helped me: there a German class there, we still have a mattress, and we still have a bike and so on.” (Cesare, 515-525)*

Administrative practice and financial support were often unclear and contributed to the stress, especially as this was combined with rising electricity and heating costs. Host also had to approach the authorities proactively and hardly ever vice-versa; contact persons at social services did not exist, changed jobs, or were not available.

Many hosts got the impression that the state did not recognize their needs of care and support. Due to the lack of institutionalization of private accommodation and the overburdening of the authorities, the hosts were left on their own, especially at the beginning.

Tronto’s category of caring about as the competence of recognizing needs allows two insights to be gained here. One is that, hosts need skills in order to recognize the needs of refugees, which is often but not always the case. The second is that, hosts also express a need for support and feel that this is not being met, pointing to a conflict at the institutional and political level in terms of civil society receiving appropriate recognition or compensation for their services. It refers to the moral dimension of caring and reminds the state to assume, and to take seriously, responsibility for their needs.

### 5.3 Caregiving—practical dimension of care

In the online sample and the interviews, meeting the caring needs was a very important issue. Organizing care means planning, coordination, and doing diverse organizational tasks. Hosts were constantly weighing up and assessing situations: how would the household have to be reorganized? How much help would they be prepared to give? How to deal with the sudden arrival of other family members? What should they do if the Ukrainians were unable to find their own apartment within a reasonable period of time? Others sought advice from a psychologist beforehand on how to deal with traumatized people.

The degree of support and care varied. The qualitative interviews showed that some hosts and refugees were in a relationship that was more reminiscent of a classic tenancy, especially when refugees lived in separated parts of the house, without any further aspects of care. Hosts just provided furniture or household items, and after moving in there was no close contact between the hosts and the refugees. However, this situation was a minority. In the online survey 90% of the hosts declared support with everyday issues like waste sorting, internet access, or shopping. Over 60% declared support with health issues, and 62% supported refugees in applying for social assistance benefits. This may have included accompanying them to visits with social services or organizing a translator. Finding language courses was provided by almost 60% of hosts.

Hosts in the survey occasionally mention supporting the refugees with cash because social assistance is so low.^7^ For some, compensation from the authorities for incidental costs (energy, water) was still unclear and they mentioned financial burdens.

Other hosts spent “surprisingly much time for support “(ID 603) and provided a very high level of practical support. This was shown in the interviews with the hosts. Ms. Bader, for example, responded to a hundred housing advertisements before she found an apartment for the refugees:

*“… that was a half-day job. In the morning, I looked at the advertisements, made phone calls, asked ‘do you take Ukrainians?’, some immediately said no. (…) That’s legitimate if you don’t know when they’re going home again, One day, in my Excel list, I noticed the hundredth appointment, so it really was a half-day job.” (Bader, 180-187)*

In addition to practical support, the hosts also acted as conversation partners for the people they hosted and thus provided emotional support.

Ms. Cesare hosted a woman of over 70 years old in her apartment who had had a heart attack and took blood pressure medication. She describes the great sense of responsibility she had, feeling alone with no support, especially at the beginning.

*“I thought, yes, if she dies now tonight, it’s my fault, I called someone in desperation.” (Cesare 509 - 512)*

Catia, a Ukrainian woman, appreciated the active support of their hosts. She describes that it was emotionally important for her to have people around who cared for her, both physically and emotionally:

*“And then we always had dinner together and she [“host mother”] cooked for us, which was physically and emotionally helpful because we were always kind of together, so I could pull myself together. Otherwise, I would have just locked myself in my room. If I’d been on my own, I think it would have been a lot worse, (…) So, I think it also gave me a chance to communicate. The host mom would just ask and wait for me to cry or tell, so I think that was a little bit like therapy. I think it was helpful for me and I think it was helpful for most of the people I know.” (Catia, 43-54)*

Also, Dora describes how the private accommodation helped her to integrate:

*“I am very happy that I ended up with a Swiss host family and not in a camp with other people. Not because I think they are bad, but because I realized that it means integration for me. That is, if I had come into a social circle that I really knew, where I could speak, where I knew the traditions (…) I don’t think I would have made such big steps. Everything that happened in most cases was with their help, and the speed with which it happened was also thanks to their help.” (Dora, 229-235)*

The online survey showed that many hosts and refugees shared leisure time. Among other things the open answers mentioned joint activities, networking with clubs and third parties, or inclusion in neighborhood activities. Around 45% of the hosts also supported refugees in their search for employment. These networks are highly relevant for labor market integration (Scherr and Yüksel, 2019). Almost a third of Ukrainian job seekers in Switzerland find their job through personal contacts (Strauss et al., 2023, pp. 9–11). The survey shows that female and male hosts were equally involved in this form of practical care work.^8^

To address caring needs and to get knowledge about how to care (Tronto, 1998, p. 16), communication is a very important issue.

“Good cohabitation requires mutual attention and respect from everyone” wrote one host (ID 579). Open communication and ideally a shared language facilitates caregiving and enables good relations. Mutual care, like the sharing of household chores or even cooking together also facilitates a good relationship between hosts and refugees,

*“We took in Afghans who had come from Ukraine. They had to flee Afghanistan 10 years ago and then went to Ukraine, where they built a life for themselves. (…)They cooked for us for 7.5 months and helped us with the housework and gardening.” (ID 1612)*

Some hosts described the positive relationship as familial and the rediscovery of family roles both sides agreed upon was appreciated (see also ID 108, 702): Besides, a “shared flat situation” was associated with positive dynamics—it refers to a relationship between equals:

*“We adjusted to flat-sharing life right from the start. We didn’t have any great expectations of ourselves or our flat mates. We ate together a lot, but everyone was free to live their daily life at their own pace. It was a nice way of living together. We cried and laughed together. We laughed a lot.” (ID 575)*

### 5.4 Care-receiving: negotiating power relations through care

Care relationships are also places where power relations are negotiated. This might lead to conflicts on a personal level between hosts and refugees.

#### 5.4.1 Mutual care relations

Care as mutual give and take can lead to a relationship in which the power imbalance between the refugees and the host society can be temporarily transformed. In some situations, care giving was reciprocal: Ukrainians took on practical care work, such as cooking or looking after children. Emotional care in some situations was also reciprocal through the friendly relationships that have developed:

*“I understand that they support me. I mean, on the one hand, and on the other hand, they are also there, they have their own problems, and I can support them, and I do, so there is this kind of mutual exchange. And I think that in my particular situation, both families have benefited. Although sometimes I wondered, well, what can I give them? But if you’ve built up friendly relationships, then you can give them a lot in return for their kindness. It’s a bit like that.” (Irina, 190-197)*

#### 5.4.2 Denying and rejecting care—perspective of refugees

Care also can be denied or rejected, not only by the hosts but also by the refugees, and the cycle of care is disrupted. Such situations can be full of misunderstandings and tacit expectations, as well as exhaustion and stress. At the same time, in these moments when refugees are rejecting care, the power relations that exist between care givers and care receivers are called into question.

According to Tronto, care receiving requires a complex moral element of responsiveness, because it shares the moral burden among the person that has received the care but also involves the moral attention of the ones who are doing the caring work (Tronto, 1998, p. 17). Indeed, Ukrainian interviewees described receiving care as an area of tension. This was particularly the case when they did not want to take advantage of offers of care, in order to maintain their own independence, as the following quote illustrates:

*“I’ve always bought my own food because, let’s say, I don’t like the feeling of sitting on someone’s back. I don’t like that feeling. So, I bought my own and ate my own, and I think at some point they might have even been offended that I rejected their help.” (Erna, 182-186)*

Rejecting offers of care can also affect the social level, for example if the Ukrainians did not want such close contact with the host family:

*“And they came to us all the time, of course that wasn’t pleasant, seeing them every day, doing something with them, going to events. Their children had a concert at the time, and we were simply told to come to the concert and see their child sing. (Anushka, 228-231)”*

Clearly, some refugees had a great need for peace and quiet due to the events of war and flight, so the social activities offered by the hosts overwhelmed them:

*“And when you arrive, you thank them for their hospitality, and they want to give you (…) this, or that, but you don’t want that, you just want to, I don’t know, lie under a blanket, relax somehow. And in the end, you refuse and somehow hurt the person’s feelings.” (Erna, 279-283)*

At the same time, the Ukrainian women interviewed were keen not to be a burden so as not to lose the favor of their hosts:

*“I think also for the families, for the Swiss people: how long will they tolerate us? Because they took us in and were thinking two three months until the summer and then we are not leaving. And I thought about this I mean there’s nothing I can do but I don’t want them to be upset with us and I don’t want that tension, so I thought like we do need to be more independent and to ask them less for help. When I need something, I Google it we will translate and not to bother them with every question.” (Catia, 163-170)*

These examples from the Ukrainian women show the amount of consideration and empathy for hosts which refugees need in order to navigate and sustain the relationship. But they also show that rejecting care can be a way of contesting the power relations between the host and the refugees. Rejecting care can be related to the desire for autonomy and freedom, not remaining just a helpless victim that needs care.

#### 5.4.3 Denying and rejecting care—perspective of the hosts

The hosts also reported situations in which the help offered was refused, which led to disappointment. This was the case in the following situation from the survey. A couple hosted a mother and daughter, and some months later the mother returned to Ukraine leaving the daughter in Switzerland, so they suddenly became official foster parents:

*“On the one hand, we see the situation as a good experience. The daughter has grown close to our hearts. Nevertheless, we have mixed feelings about the situation. We think it’s a shame that very little use is made of the family connection and that we sometimes have the impression that her life is being organized without us (e.g. meals). (…) This is sometimes a little frustrating but is perhaps also typical of a teenager. (…) However, we have taken in refugees with the basic attitude of doing so without expectations. There is no contact between our children and her. This will be difficult in the long term. We regret this very much and would have liked it to be different. Sometimes it’s just plain tiring to always give and get nothing (or hardly anything) in return.” (ID 647)*

In the survey, many hosts complained. They assume the power of definition and attributed passivity, unfriendliness, lack of interest in integration, a demanding attitude, and ingratitude to refugees. Yet, some expressed their regret:

*“I would have liked to play a more active part in the integration of the refugee I took in. Unfortunately, the refugee didn’t share my goal because he wasn’t interested in language classes, while I did everything I could to make him understand that a minimum level of (language) was necessary for integration and finding a job. The refugee was not interested in finding a job when he was a professional horticulturist and there were many opportunities for employment if he had been motivated to work.” (ID 379)*

Some reflected on this as a reaction to an inherently stressful life situation, combined with the suggestion that hosts should be better prepared for their task. Rarely, the hosts explicitly recognized the war situation as a major obstacle to refugee’s arrival and integration:

*“We often forget that refugees who have been bombed are very unstable and sick people. The Swiss government has not provided enough support in this case (…) This is one of the main reasons why these people cannot integrate quickly. The majority of refugees are in the deepest and most severe depression, that our Swiss medical professionals have never seen before, and we want them to learn the language and go to work.” (ID 914)*

There are also some examples of a paternalistic attitude from the host, of expecting the refugees to fulfill their requirements and conditions.

*“Our most important/only expectation was that he would make an effort to improve his (…) language skills (the accommodation contract was linked to this). We financed a German course for him for this purpose (25 lessons), but he only attended 5 and then didn’t continue despite reminders (one reason why we cancelled the housing). To make matters worse, he rarely/never communicated if and when he was at home and, for example, wanted to eat with us, etc., and was practically uninvolved in the household. (…) Over time, we lost interest in each other.” (ID 529)*

This example reflects the official integration discourse of promoting and demanding, in which support is tied to conditions.

These examples demonstrate that to care about requires some competence from the hosts, *“listening to articulated needs, recognizing unspoken needs, distinguishing among and deciding which needs to care about.”* (Tronto, 1998, p. 16). This is especially the case regarding the need to rest linked to the possible experience of trauma. It shows that homestay accommodation means more than just providing a room, but rather needs professional preparation and support to understand the needs that must be cared for.

### 5.5 Caring with—beyond the temporary

According to Tronto (2013), this dimension of care refers to processes that evolve over time. This dimension is of particular interest in the context of homestay accommodation. Although long-term studies are lacking, there are some promising indications that homestay accommodation can facilitate the social integration of refugees by building social capital (Strauss et al., 2023). However, many statements from host families suggest that private accommodation can become more challenging over time. Ultimately, those who have been taken in do not want to be a burden on their hosts. Depending on the layout, homestay accommodation is therefore only appropriate for a limited time. Maintaining privacy over a longer period is probably the biggest challenge for both sides, as in many cases the existing living environments are not designed for this.

*“But after six months, the two ladies are still with us. We need to work out an exit strategy. The two ladies need to be able to stand on their own two feet and find somewhere a bit bigger, and we would like to have our bedroom back and a certain nonchalance in the house.” (ID 880)*

The available living space also determines the opportunities for privacy, rest and relaxation. Lack of space and conflicting usage requirements can be a source of stress, for example if there is a “traffic jam” in the only bathroom in the morning because everyone, including the refugees, is heading out to work.

## 6 Discussion

Tronto’s model of different phases of care proved useful to analyse care in homestay accommodation. Caring about reveals the importance of becoming aware of refugees needs. This implies first of all the motivation and willingness of persons from civil society to offer space in their residences to refugees. Further studies are needed to analyze the willingness of persons to offer homestay accommodation in the long run. Second it is important that the real needs of the refugees are identified and then taken care of. To provide this moral and ethical quality of care requires specific competences. Hosts should not be left alone with their caring responsibilities, but rather embedded within professional support.

Supporting the hosts can also be important in order to enhance and ensure their capacities of caring for. Considering the phase of caring for (Tronto, 1998) allows us to ask who is ultimately responsible for organizing the identified care, and for marshaling the relevant resources and personnel. This question is linked to the relations and division of responsibilities between the state, civil society, and private households. The answers of the hosts in our sample clearly show that they identified a need for more support, stating that they are not willing to take over the responsibility for accommodating refugees on their own.

This question is linked to “caregiving” (Tronto, 1998) and the ability of individuals and organizations to perform the necessary caring tasks: “Incompetent care is not only a technical problem, but a moral one” (Tronto, 1998). Who then is responsible if hosts are not able to meet the caring needs? The care work provided by hosts is embedded in a migration policy context and a legal framework that defines what needs are, and how much care might be provided for refugees. Some hosts express their moral duty to go beyond the care provided and supported by the state, and others address inequalities in the legal framework, as there is a difference in care supported by the state for the different categories of refugees.

Analyzing the phase of care receiving has shown different responses to the care that has been given. Refusing care or accepting it as temporary support is one way for refugees to maintain their autonomy and independence, and to challenge power asymmetry. In a few cases the mutual provision of care can lead to existing power relationships being changed, and a relationship on an equal footing being established. Conceptualizing care as a reciprocal relationship is one way to temporarily establish a symmetry of power.

Last but not least, Tronto (1998) conceptualization helps to analyze the power relations that are involved in the care process. Care in homestay accommodation reflects the existing power asymmetry between refugees in precarious situations and the established hosts who have plentiful resources, both in terms of living space and time.

Hosts have the power to terminate the private accommodation and send, for example, refugees back to collective shelters. Refugees can withdraw from or deny care as a strategy to react to offered care that might not fit with their needs. Finding housing on their own can also be an option, however this might be quite difficult for refugees regarding the requirements of the housing market.

If refugees’ needs are truly perceived and understood, they receive the protection they need in the initial period, and they also gain access to the host society which could be identified as (limited) a form of power sharing.

At the same time, the hosts also have needs that are not being met. This shows a tension in the question of responsibility for the reception and care of refugees. On the one hand, civil society is prepared to support and supplement the state, but at the same time they are not prepared to provide these services free of charge in the long term. Civil society can also show solidarity with refugees to help improve their situation vis-à-vis the state.

The hosts’ commitment and their demand for more state support can be seen as a sign of solidarity with the refugees vis-à-vis the state and, to some extent, as a criticism of the state. It challenges the division of responsibilities and duties between the state and civil society, Remarkably, no formal association for homestay accommodation or the interests of hosts has been formed to date unlike in other European countries—«caring with» seems not to be an interesting option, and the field is left to well-established professional actors. In addition, however, there are also statements by the hosts that show that they will pass on the state’s demands to the refugees. This is evident in the so-called paternalistic statements of the host families, which make demands on the refugees. If these demands are not met this can lead to disappointment, which in turn hardens (existing) prejudices against refugees, and can lead to the relationship breaking down.

As conflicts in homestay situations are inevitable and connected to space, privacy, and communication this form of accommodation s better suited to short-term stays. Ample living space and separate facilities, on the other hand, are better suited to long-term stays. Social Democrat Samira Marti initiated a Parliamentary postulate if the experiences from the war in Ukraine can result in a continuation of private accommodation. Therefore, the State Secretariat of Migration has commissioned a report including a cost–benefit analysis of homestay accommodation.^9^

## 7 Conclusion

This analysis shows the various dimensions and amounts of care work that hosts do. Practical and emotional support often goes hand in hand with a large mental load, especially when responsibilities have not (yet) been clarified, or the placement is not (yet) institutionalized. In this way, civil society is making a major contribution to the accommodation of refugees and relieving the state of part of its burden.

Focusing on care in homestay accommodation allows us to reveal power imbalances between hosts and refugees, as well as strategies how to come to terms with the situation. Hosts have the power to define who, for how long, and whether and how they facilitate the social integration of refugees. The withdrawal of refugees or the rejection of offered care can be understood as a strategy to distance themselves from this assigned role as victims and recipients of help. However, denying care in a situation of power asymmetries also poses a considerable risk to refugees.

The wider institutional and political context adds to this landscape of power imbalance. For example, the S status is temporary, at time of writing in December 2024, Parliament has decided on severe, yet hard-to-implement, restrictions on getting status S.^10^ A recent study of the Canton of Zurich revealed that all categories of refugees face similar challenges and burdens: living in collective shelters in remote areas, having scarce financial resources, and finding it challenging to search for jobs (including finding affordable childcare), learning the language, and having uncertain prospects of staying in Switzerland (Eser Davolio et al., 2024, p. 55). Homestay accommodation has the potential to build important networks for the integration of refugees. If hosts are ready to identify and provide the care needed, refugees can profit from the networks of their hosts, who are usually well established in society and therefore possess valuable resources and connections to facilitate integration processes.

The high level of commitment shown by civil society in 2022 poses the question of to what extent this commitment will continue and can be established as a standard way, among others, of refugee reception. Anecdotal evidence from other countries suggests that the willingness to provide home stay accommodation follows cyclical trends and is currently in decline. Our results suggest that this may depend in particular on how host families are supported in their ability to identify and meet the effective needs of refugees, and to mitigate power imbalances. This is linked to the question of the division of tasks and responsibilities between the state and civil society. Meanwhile, the Swiss Refugee Council has set up a program to prepare and support prospective hosts which both cantons and municipalities can use, and which addresses the well-known pitfalls of long-term hosting.^11^

The refugees interviewed all arrived in Switzerland in the spring of 2022, shortly after Russia started the war against Ukraine. At this time protection status S was newly introduced, and the structures for accommodating refugees were only just being established and expanded. The uncertainties and ambiguities often mentioned in connection with residence status and support options could be linked to this. Depending on the canton and municipality, private accommodation has since been better regulated and in some cases corresponding support structures have been established.

Support for Ukrainian refugees in Switzerland remains high (Sotomo, 2023, p. 20). In late 2023, the majority favored equal conditions for refugees, regardless of their home country (Sotomo, 2023, p. 23). State recognition and support of homestay accommodation could help make it an additional standard form of refugee reception. Necessary conditions for this to occur include public support and supervision of hosts, as well as networking and training opportunities. Recognizing this engagement by civil society would also result in appropriate and uniform financing, as well as accessible, clear, and reliable communication from the authorities to help ease the mental load of hosts (Ammann Dula et al., 2024, p. 4).

Follow-up research is necessary to get a more nuanced account of the long-term impact of homestay accommodation on social integration. This should include a detailed analysis of forms of civic engagement in this sector.

## Data Availability

The raw data supporting the conclusions of this article will be made available by the authors, without undue reservation.

## Footnotes

^1^See Bassoli and Luccioni (2024) for the term.

^2^ https://www.sem.admin.ch/dam/sem/de/data/integration/foerderung/programm-s/rapport-programm-s-2024.pdf.download.pdf/rapport-programm-s-2024-d.pdf

^3^The information in this section is mainly taken from Ammann Dula et al. (2024), p. 3–4.

^4^See https://www.bfs.admin.ch/bfs/en/home/statistics/cross-sectional-topics/housing-switzerland.html

^5^Following the Parliamentary postulate 23.3203 Samira Marti (Social Democrats) on “Experiences from the war in Ukraine. Evaluation of the integration and savings potential of a continuation of private accommodation in the asylum system” https://www.parlament.ch/de/ratsbetrieb/suche-curia-vista/geschaeft?AffairId=20233203. The publication of the report is scheduled for late 2025.

^6^ https://www.ors-group.org/ors-ch-de

^7^According to the law, it has to be lower than social assistance for locals (about CHF 1000 per month) and ranges from about 350 to 850 CHF, depending on the canton.

^8^In the online survey, one person identified themselves as non-binary.

^9^Postulate 23.3203 Experiences from the war in Ukraine. Evaluation of the integration and savings potential of a continuation of private accommodation in the asylum system, cf. https://www.parlament.ch/de/ratsbetrieb/suche-curia-vista/geschaeft?AffairId=20233203. The publication of the report is scheduled for late 2025.

^10^ https://www.swissinfo.ch/ger/r%C3%A4te-wollen-status-s-nicht-mehr-f%C3%BCr-alle-ukrainischen-gefl%C3%BCchteten/88438870

^11^ https://www.fluechtlingshilfe.ch/aktiv-werden/gastfamilien
